# The Role of Decorin Proteoglycan in Mitophagy

**DOI:** 10.3390/cancers14030804

**Published:** 2022-02-04

**Authors:** Thomas Neill, Renato V. Iozzo

**Affiliations:** Department of Pathology, Anatomy and Cell Biology and the Translational Cellular Oncology Program, Sidney Kimmel Cancer Center, Thomas Jefferson University, Philadelphia, PA 19107, USA

**Keywords:** small leucine-rich proteoglycans, autophagy, Peg3, VEGFR2, MET, mitostatin

## Abstract

**Simple Summary:**

The eminent rise of extracellular matrix constituents, chiefly hailing from the proteoglycan gene family, has revolutionized our understanding of how intracellular catabolism is regulated at the intersection of autophagy and breast cancer. In this review, we examine the mechanisms of decorin, a small leucine-rich proteoglycan, as it relates to autophagy and mitochondrial autophagy (mitophagy). In each case, decorin signals via a unique cell surface receptor tyrosine kinase to evoke autophagy (VEGFR2) or mitophagy (MET receptor) that converges on a novel tumor suppressor gene. The downstream function of either Peg3 or mitostatin in response to decorin manifests as potent means to subdue breast cancer development and progression.

**Abstract:**

Proteoglycans are emerging as critical regulators of intracellular catabolism. This rise in prominence has transformed our basic understanding and alerted us to the existence of non-canonical pathways, independent of nutrient deprivation, that potently control the autophagy downstream of a cell surface receptor. As a member of the small leucine-rich proteoglycan gene family, decorin has single-handedly pioneered the connection between extracellular matrix signaling and autophagy regulation. Soluble decorin evokes protracted endothelial cell autophagy via Peg3 and breast carcinoma cell mitophagy via mitostatin by interacting with VEGFR2 or the MET receptor tyrosine kinase, respectively. In this paper, we give a mechanistic perspective of the vital factors underlying the nutrient-independent, SLRP-dependent programs utilized for autophagic and/or mitophagic progression in breast cancer. Future protein therapies based on decorin (or fellow proteoglycan members) will represent a quantum leap forward in transforming autophagic progression into a powerful tool to control intracellular cell catabolism from the outside.

## 1. Introduction

In spite of the significant advances in breast cancer diagnosis and treatment, this malignant neoplasm is still the most common cancer diagnosed among women and represents the second leading cause of cancer mortality in the United States after lung cancer [1]. One of the most striking features of mammary carcinomas is their heterogeneity both in terms of tumor cell types and the stroma, an associated tissue long considered an active participant in malignant behavior, metastatic spreading, and colonization of distant organs [2,3,4,5,6]. Indeed, intratumor heterogeneity has been proposed to represent the “*Rosetta Stone*” of therapy resistance [7]. This concept is based on the idea that acquired tumor resistance to targeted therapies depends on intratumor heterogeneity and diversification during the therapeutic process enabling cancer cells to escape death [7]. There is emerging evidence that breast cancer progression, metastasis, and treatment resistance may depend on intratumoral molecular subtypes and their interconversion among seemingly different subtypes [8]. Moreover, differences in diffusion and consumption rates of growth factors and cytokines would contribute in modulating the microenvironment, further promoting phenotypic heterogeneity [7]. Accordingly, both survival and recurrence rates in mammary carcinomas are variable and the biological underpinnings that affect clinical outcomes need to be fully elucidated [5].

There is a mounting body of evidence pointing to the breast cancer stroma as a key regulator of tumor progression after the initial stages of tumor formation, and that the tumor stroma may also contribute to chemoresistance [9]. For example, stromal cells can extrinsically alter tumor cell drug responses with profound consequences for patient outcome [9]. Thus, aspects of stromal biology, including mesenchymal, stromal, immune cells, and cancer-associated fibroblasts, are important to fully understand the molecular and cellular mechanisms contributing to breast cancer development and progression. Proteoglycans are key constituents of the breast cancer stroma and mediate many important functions including angiogenesis, growth factor sequestration and presentation to various receptors, immune modulation, and autophagy, among many other roles [10]. 

In this review, we critically assess the role of proteoglycans, especially focusing on decorin, in autophagy and mitophagy and propose a new paradigm whereby soluble extracellular matrix constituents with biological activity significantly affect intracellular catabolic events linked to breast cancer progression and metastasis.

### Proteoglycans Are Versatile and Emergent Autophagic Regulators

As a ubiquitous and genuinely multifunctional entity necessary for maintaining homeostasis, the extracellular matrix (ECM) is a reciprocal dynamism of highly interconnected and interacting macromolecules that nourishes and encapsulates all cells within tissues and organs [11]. Proteoglycans (PGs) represent a major class of these versatile ECM molecules. They exert not only a structural and architectural role within their tissue of residence, but are chief signaling effectors responsible for controlling key facets of cellular behavior in response to ever-changing stimuli [12]. To date, there are about forty-three genes that encode proteoglycans, with many variants postulated to exist due to alternative messenger RNA (mRNA) splicing to further fine tune their function in a diverse array of cell types and tissues [12]. Proteoglycans (PGs) are subject to heavy post-translational modifications, such as their hallmark motif that acts to differentiate this class from other ECM molecules, the covalent attachment of one or more glycosaminoglycan (GAG) chains to the protein core. These chains come in four common flavors, chondroitin sulfate/dermatan sulfate, keratan sulfate, and heparan sulfate [12]. Additionally, glycosylation events also occur to generate *O*- and *N*-linked oligosaccharides that further decorate the protein core. The GAG chains are frequently sulfated, which generates a code for the binding, sequestration, and release of various growth factors; this is especially critical for establishing morphogen gradients during development and has implications in disease [13].

Proteoglycans are highly dynamic molecules involved in a plethora of homeostatic cellular processes, ranging from initial modeling and subsequent remodeling of local and organismal ECM architecture, bi-directional cellular signaling, tissue repair, development, inflammatory responses, proliferation, migration, and varied immune responses [13,14,15,16,17,18,19]. However, PGs are functionally relevant in cancer biology via their innate ability to regulate angiogenesis and induce autophagy (see below) within the breast tumor stroma and mitophagy in the parenchymal cancer cells [13,20,21,22,23,24,25]. The highly coordinated mechanisms of action of PGs in cancer depend on direct interactions with cell surface receptor tyrosine kinases (RTKs), such as MET and vascular endothelial growth factor receptor 2 (VEGFR2), integrins, and Toll-like receptors that are expressed by stromal cells, breast cancer cells, and macrophages [20,26,27,28].

Autophagy is an essential and evolutionarily conserved homeostatic process where various organelles (superfluous, damaged, or aged) and/or cytosolic components (protein aggregates, foreign nucleic acids) are degraded and recycled via lysosomes [14,23]. It must be noted that the role of autophagy in regulating cancer progression has met with a substantial amount of controversy. This is warranted, as initial reports pointed to an almost exclusive pro-tumorigenic and pro-survival function, as the catabolism of intracellular compartments enhances cell survival through periods of nutrient scarcity, until the angiogenic switch is engaged [29,30]. However, new and mounting evidence proposes that inducing or augmenting autophagic activation within cancer cells and their surrounding stromal cells can lead to tumor cell death, reduce malignant angiogenesis, and impede local and distal metastases to lymph nodes and organs [31,32,33,34]. Collectively, an important functional paradigm in cancer biology has emerged at the intersection of autophagy and angiogenesis. Proteoglycans and, perhaps other ECM molecules that possess pro-autophagic tendencies, can be categorized into two bins [35,36]. That is, the PGs can either be anti-angiogenic and pro-autophagic, i.e., decorin (see Section 2), or those exhibiting strong pro-angiogenic effects that will simultaneously inhibit autophagy, i.e., perlecan [37,38,39,40]. Therefore, we postulate that proteoglycans are heterobifunctional, and depending on the cell context and/or expression level, can inhibit or enhance autophagy [24,41]. Therefore, in this Review, we examine the role of decorin in orchestrating and evoking mitochondrial autophagy within breast cancer. Indeed, protein therapeutics that can leverage this form of latent bivalency would make for potent therapies in the ongoing fight against cancer.

## 2. Decorin Is the Prototypical Heterobifunctional Small Leucine-Rich Proteoglycan

Intracellular signaling events that are mediated by PGs are primarily evoked by the binding of soluble, extracellular proteoglycans to their cognate receptor to enthusiastically modulate cell homeostasis by controlling downstream signaling cascades. Decorin derives its eponym for its ability to specifically bind periodic collagen type I [42,43,44], and functions not only as a “collagen decorator”, but also as an important regulator of collagen fibrillogenesis both in vitro [45] and in vivo [46,47,48,49,50]. The genetic ablation of the *Dcn* gene causes a skin fragility phenotype [51]. Decorin was originally discovered by several laboratories and designated DSPG1 or PG40 because of its apparent molecular weight of the protein core [52] and subsequently identified in various tissues [53,54] and in the stroma of colon cancer [2,55]. Decorin has been utilized as an anti-fibrotic agent because of its ability to bind many isoforms of transforming growth factor beta (TGF-β [56,57,58,59,60], thereby sequestering this powerful growth factor in the pericellular matrix. The lack of decorin in various mouse models of mesenchymal and epithelial neoplasms is permissive for tumorigenesis [61,62,63]; conversely, decorin can suppress tumorigenesis, invasion, and metastasis of inflammatory breast cancer [64]. This is further underscored by a recent study demonstrating that decorin is downregulated in senescent fibroblasts, which additively drives the tumor-promoting phenotype of ionizing radiation induced premature senescence [65]. Decorin may also serve as an important diagnostic biomarker for patients with advanced stage (II or III) breast cancer as it emerged as an independent predictive factor for these stages [66]. Decorin is a prime mechanistic example of how PGs can elicit dramatic responses within cells via RTK signaling. Decorin is the archetypical member of the small leucine rich proteoglycan (SLRP) gene family and harbors a single covalently attached dermatan/chondroitin sulfate chain at its N-terminus. Decorin engages, with a hierarchal affinity, various RTKs, including epidermal growth factor receptor (EGFR) [67,68,69,70], MET [71] and VEGFR2 [20,72,73]. Intriguingly, the GAG chain that decorin possesses appear to be dispensable for many, if not all, of the below discussed functional activities [71,72,74,75,76]. Indeed, it appears the GAG chain is required for the proper spacing and alignment of type I collagen fibers during fibrillogenesis and overall matrix organization [77,78,79].

In target-rich environments, such as those found on the surface of breast cancer cells, upon decorin binding, the RTK undergoes dimerization, a rapid burst of phosphorylation occurs on the intracellular tails, and finally internalization and consequent lysosomal degradation of the decorin/receptor complex [72,80,81]. Via this mechanism of action, decorin is potently anti-angiogenic [28,82] by suppressing *HIF1A* expression in a non-canonical manner and inhibiting the synthesis and release of intracellular and secreted vascular endothelial growth factor A (VEGFA) [74]. Simultaneously, decorin promotes the expression and rapid release of potent anti-angiogenic effectors, such as thrombospondin-1 [83]. However, this was only a chapter in the much larger novel that is the story of decorin [81], an oncosuppressive molecule with a high potential for becoming an adjuvant therapy for human epithelial malignancies [84].

### 2.1. Decorin Is a Soluble Pro-Autophagic Tumor Repressor

The hypothesis of autophagic induction as oncosuppressive [85] is underscored by critical genetic experiments demonstrating an increase in tumor burden and progression, following the heterozygous deletion of *Becn1*, which encodes Beclin 1, a core autophagic component [86,87,88]. A deeper (and perhaps much more relevant line of evidence for autophagy as anti-tumorigenic and its relationship to decorin) explicitly involves an RTK-dependent mechanism. This originates from the finding that EGFR, a target that decorin potently suppresses [67], avidly phosphorylates and inactivates Beclin 1 [89] via Akt [90]. In this manner, EGFR suppresses Beclin 1, leading to increased chemoresistance and tumor progression [89]. The converse also holds where augmented autophagy suppresses HER2-mediated tumorigenesis [91]. One of the key properties of decorin is the differential regulation of RTK trafficking [71]. This is exemplified by distinct populations of either decorin/MET or hepatocyte growth factor (HGF)/MET, where decorin triggers the association of MET with caveolin positive endosomes for degradation whereas HGF promotes interactions with clathrin for sustained recycling of MET to the plasma membrane for continued oncogenic signaling [71]. It was thought that decorin promoted internalization and degradation via lysosomes in this manner for both EGFR and MET (and may represent a general mechanism for decorin bound RTKs); however, perhaps it is via autophagic degradation as LC3C can mediate MET trafficking in response to autophagic signals [92]. As an additional layer of regulatory complexity, we found that nutrient deprivation, a classical signal for autophagic induction, triggers the expression of decorin mRNA and protein in murine cardiac tissue [24,41].

**Figure 1:**
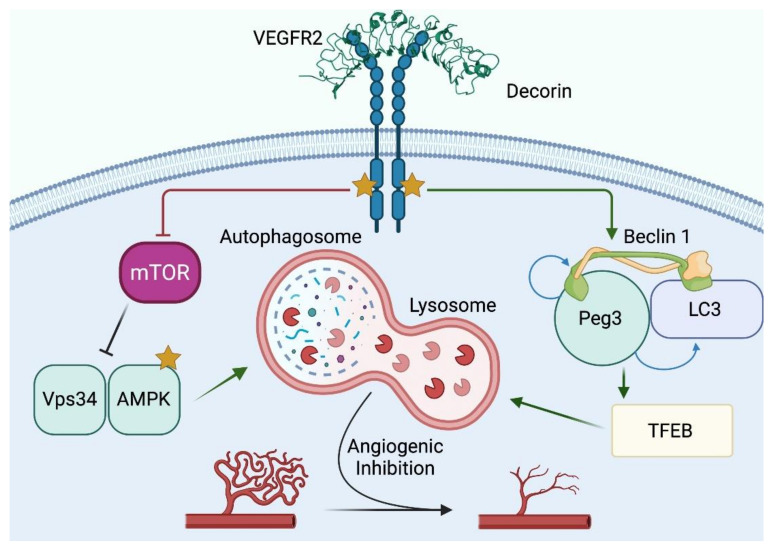
Schematic depiction of decorin-evoked autophagy in endothelial cells. The PDB accession ID for decorin is 1XCD. Please consult the manuscript for additional details. Images generated using Biorender. Abbreviations used: VEGFR2, vascular endothelial growth factor receptor 2; mTOR, mechanistic target of rapamycin; Vps34, vacuolar protein sorting 34; AMPK, AMP-activated protein kinase; Peg3, paternally expressed gene 3; LC3, microtubule associated protein 1 light chain 3; TFEB, transcription factor EB.

**Figure 2:**
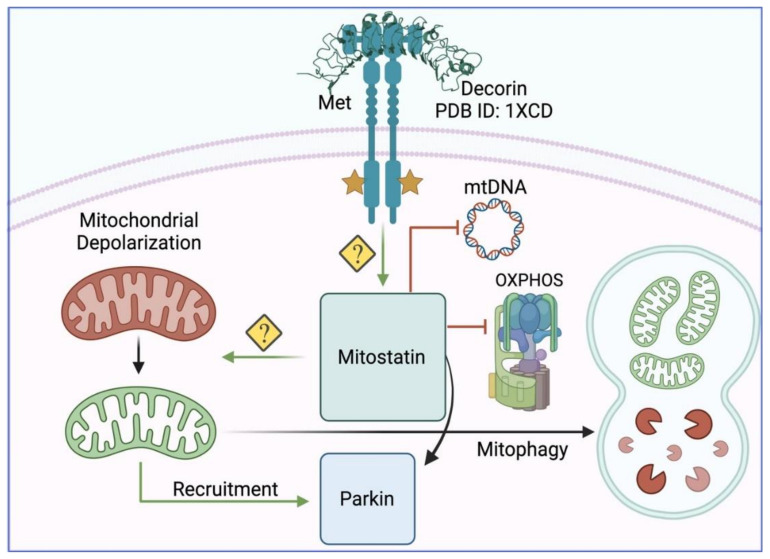
Schematic representation of decorin-evoked mitophagy in triple negative breast carcinomas cells. Please consult the manuscript for additional details. Images generated using Biorender.

We discovered that nanomolar amounts of soluble, monomeric decorin [93] evokes protracted and non-canonical endothelial cell autophagy (Figure 1) [72] and breast cancer cell mitochondrial autophagy (mitophagy) [80], directly within the tumor parenchyma (Figure 2). Thus, decorin concurrently targets distinct histological compartments, whose specificity is determined by the type of cell surface RTK expressed. Indeed, decorin binds VEGFR2 expressed by the endothelial cells and MET that abundantly (that is, target-rich) adorns breast cancer cells. In this manner, decorin triggers the formation of bubble-like structures in endothelial cells reminiscent of autophagosomes. These structures, originally detected by differential interference microscopy, were morphologically validated by co-immunostaining for Beclin 1 and microtubule associated protein 1 light chain 3 (LC3), two key autophagic effectors [94]. This discovery positioned decorin as the first soluble SLRP capable of evoking autophagy.

### 2.2. Decorin Evokes Endothelial Cell Autophagy and Mitophagy

Before delving into the discovery of decorin-mediated mitophagy in breast cancer, we will briefly review the general mechanism of decorin-evoked autophagy in endothelial cells as a starting point (Figure 1). In genetically stable primary cultures of endothelial cells, decorin binds the ectodomain of VEGFR2 at IgG_3-5_, which partially overlaps with VEGFA binding (IgG_1-3_) [72]. This high-affinity decorin/VEGFR2 interaction results in the rapid activation of the α catalytic subunit of AMP-activated protein kinase (AMPK), the master energy sensor kinase that has been previously implicated in cancer inhibition [95]. AMPK regulates a plethora of intracellular catabolic processes, including autophagic initiation. The conventional AMPK activation follows from times of cellular stress, e.g., a nutrient dearth where the AMP/ATP ratio is significantly elevated, to induce autophagy [96]. This is in stark contrast to the non-canonical mechanism utilized by proteoglycan-mediated autophagy, which occurs in an RTK-dependent manner and in nutrient-rich conditions where the AMP/ATP ratio is conducive to normal physiological function [72]. Silencing VEGFR2 via RNAi strategies or small molecule inhibitors to pharmacologically impair the VEGFR2 kinase, abrogates decorin signaling and thus impairs autophagy [72].

From a top-down or outside-in [23] signaling perspective (decorinαVEGFR2α autophagosome), autophagy initiates from a discrete subcellular region referred to as the phagophore assembly site (PAS). The molecular composition of the PAS is known to contain the p110 class III (non-oncogenic) PI3K vacuolar protein sorting 34 (Vps34), human Unc-51 Like Autophagy Activating Kinase 1/2 (hULK1/2), Atg13, and FAK-interacting protein of 200 kDa (FIP200) [97,98]. Decorin requires Vps34 and induces phosphorylation of AMPK at Thr^172^ [99] (Figure 1). Inhibiting Vps34 with 3-methyladenine (3-MA) or AMPK with Compound C (Dorsomorphin) abrogates decorin-mediated autophagy [72]. AMPK opposes mechanistic target of rapamycin, complex 1 (mTORC1), which is responsible for fundamental anabolic pathways coordination cell growth, cell size, and proliferation [100], thereby making mTOR staunchly anti-autophagic. Decorin attenuates the mTOR axis (Figure 1), by decreasing phosphorylated mTOR at Ser2448, Akt at Ser476, and p70S6K at Thr389 [99].

These signaling cascades results in a specific pro-autophagic signature written in the language of protein phosphorylation. At its terminus, this signature converges on the expression and cytosolic accumulation of Peg3 (Paternally expressed gene 3) [72]. *Peg3* was identified from a subset of differentially expressed genes exclusively within the murine tumor stroma of triple negative orthotopic tumor xenografts treated systemically with human recombinant decorin [72,75,101]. Intriguingly, since Peg3 non-canonically disrupts Wnt/β-catenin signaling [102], in a mechanism akin to how decorin suppresses β-catenin downstream of MET [71], we pursued *Peg3* as a candidate gene. *Peg3* encodes a genomically imprinted, Krüpple-like zinc finger-containing transcription factor. Initially characterized as a tumor suppressor [103,104], we discovered that Peg3 acts as a nexus for decorin- (and other PGs [105,106])-mediated autophagy [72,80] (Figure 1). Peg3 associates with autophagosomes in human and murine microvascular and macrovascular endothelial cells via co-localization with Beclin 1 and/or LC3 following decorin as a stimulus [72]. Mechanistically, Peg3 is necessary as it is required for promoting *BECN1* and *MAP1LC3A* expression downstream of decorin/VEGFR2 signaling [80]. Importantly, Peg3 is also sufficient [72,107] insofar as maintaining basal *BECN1* expression levels. Therefore, Peg3 acts as a master switch for *BECN1* that not only ensures appropriate physiological levels of *BECN1* mRNA, but to also augment its expression (in parallel with *MAP1LC3A*) when the cell confronts a stimulus from outside.

A key hallmark of autophagy comes from the flux of cargo through the pathway. Measuring flux is achieved by Bafilomycin A1 (BafA1) or chloroquine (CQ), which inhibits autophagosomal fusion with a lysosome. Using these inhibitors, we discovered that decorin, via Peg3, drives autophagic flux above basal levels, resulting in excessive endothelial cell autophagy [107].

Part and parcel with driving this newly augmented autophagic flux is the Transcription Factor EB (TFEB). TFEB recognizes and binds to coordinated lysosomal expression and regulation (CLEAR)-box sequences present in the proximal promoters of many autophagy and lysosomal genes necessary for long-term (transcriptional control) autophagy [108,109,110,111]. Long-term autophagic progression is a key characteristic of decorin. Mechanistically, TFEB is kept inactive via mTOR, thus enabling cytosolic sequestration by 14-3-3 scaffolding proteins [110,112,113]. However, following an appropriate autophagic stimulus, TFEB is rapidly dephosphorylated by calcineurin and translocates into the nucleus where it promotes gene expression necessary for sustained autophagy [111]. Congruent with the long-term effects of decorin activity, TFEB is regulated downstream of VEGFR2- and in a Peg3-dependent manner [114] (Figure 1). Decorin attenuates mTOR signaling and promotes nuclear translocation of TFEB [114]. Inhibiting VEGFR2, AMPK, or using RNA to silence Peg3 is enough to inhibit decorin-mediated TFEB expression as well as its nuclear translocation. These events decrease the levels of critical lysosomal genes and reduces overall autophagic flux evoked by decorin. Conversely, increasing the amounts of Peg3 drives TFEB expression (and subsequent translocation) in a proportional and saturable manner, indicating direct promoter interactions. Therefore, Peg3 functions as a novel upstream regulator of TFEB [115] and positions TFEB as a prominent downstream transcription factor within the mechanistic framework of the decorin/VEGFR2/AMPK/Peg3 axis [114].

It is well established that decorin is anti-angiogenic (see Section 2) [74,83,116,117] that also possesses pro-autophagic functions. A new chapter concerning the functional interconnections between suppression of angiogenesis and pro-autophagic properties of decorin is emerging [26]. This chapter of decorin has been written by evaluating the intracellular degradation of VEGFA in endothelial cells via autophagy [118]. Decorin-evoked VEGFA catabolism proceeds in an mTOR-independent manner but depends on Peg3. In an observation akin to *BECN1* and *TFEB*, Peg3 is necessary and sufficient for VEGFA degradation in LC3^+^ autophagosomes. Moreover, VEGFA serves as a basal autophagic substrate as determined by assaying autophagic flux with BafA1, CQ, or transient *ATG5* silencing. Interestingly, we identified RAB24, a small GTPase that regulates basal autophagy [119,120,121], as necessary for the degradation of VEGFA following decorin stimulation. Importantly, starved mice show a substantial clearance in both aortic and cardiac VEGFA that was rescued by systemic CQ administration. This study began unifying the metabolic control of intracellular VEGFA via autophagy in response to decorin as well as other traditional, pro-autophagic stimuli, such as starvation and AMPK mimetics, such as 5-aminoimidazole-4-carboxamide-1-β-D-ribofuranoside (AICAR).

Therefore, our working model includes that the VEGFR2/AMPK/Peg3/TFEB axis is capable of decoding decorin and integrating the anti-angiogenic and pro-autophagic information encoded therein to inhibit tumorigenesis and stymie inappropriate neovascularization.

## 3. Decorin Evokes Breast Cancer Cell Mitophagy via Mitostatin

Our knowledge regarding the molecular foundations of mitophagy in mammalian cells is rapidly increasing, although it is still incomplete. It is becoming evident, however, that pro-mitophagic pathways are closely linked to the metabolic rewiring of cancer cells and their high bioenergetic demands [122,123]. There is also mounting evidence that mitophagy modulators overlap with cell cycle control and survival pathways, including those occurring after cell detachment from its ECM, migration, and metastasis [124]. Moreover, mitochondria-targeted redox agents selectively induce mitophagy in a breast cancer cells and could represent valuable therapeutic strategies to target mitochondrial metabolism in cancer [125].

As we have discussed above, decorin manifests specific cellular outcomes as dictated by the expression and differential binding to different RTKs. This mechanistic paradigm is aptly illustrated by the observation that decorin evokes mitophagy in triple negative breast cancer (TNBC) cells via MET [76]. Decorin synchronizes a concerted suppression of key mitochondrial respiratory chain subunits, from all five complexes, in conjunction with several established mitophagy biomarkers, such loss of mitochondrial DNA (mtDNA) and voltage dependent anion channel 1 (VDAC1) [76] (Figure 2). Akin to endothelial cell autophagy, decorin-evoked mitophagy occurs independently of the prevailing nutrient conditions and bioenergetic demands of the cell. Instead, it depends on MET and mitostatin, thereby manifesting as a non-canonical, receptor-mediated induction of mitophagy in TNBC cells.

### 3.1. Mitostatin Is a Tumor Suppressor Gene That Regulates Mitochondria

Mitostatin is a tumor suppressor gene known by several alternate aliases, including trichoplein, a keratin filament binding protein (TCHP). The locus physically encoding *TCHP* is located at 12q24.1 and was originally named *Ts12q*, for Tumor suppressor at 12q. However, the resulting protein translated from mature *TCHP* mRNA was renamed mitostatin, for mitochondrial protein with oncostatic activity, to more accurately reflect its primary cellular function [126]. Empirical biochemical evidence for the existence of *TCHP* splice variants is currently lacking. However, performing a deep bioinformatics search did yield the existence of a computationally predicted splice form of *TCHP* that is approximately half the size of full-length mitostatin. This predicted isoform is missing its C-terminal half, with the shared N-terminal half perfectly aligning with that of full-length mitostatin. Immunoblotting with an antibody that recognizes an N-terminal epitope across a variety of different cell types did reveal a recognized protein product that was half the size of full-length mitostatin in a variety of cell lines (unpublished observations).

Mitostatin was discovered via subtractive hybridization of cDNA libraries as an decorin-inducible gene [126]. Mitostatin mRNA and protein is differentially expressed in many tissues and is conserved across multiple species [126]. In breast and bladder cancer, mitostatin expression is frequently decreased, wholly lost, and/or exists as a mutated protein variant [127,128]. Thus, mitostatin may function as a putative tumor suppressor gene. Further evidence for this assertion comes from rescue experiments where restoration of wildtype mitostatin in prostate cancer cells significantly prevents invasive phenotypes [128]. Remarkably, this finding was faithfully replicated in two TNBC cell lines where migration in 2D and 3D substrates was significantly impaired (unpublished).

Immunostaining for mitostatin reveals a punctate cytosolic pattern with a strong mitochondrial co-localization when using mitochondrial-tagged fluorescent protein probes [128]. Biochemical fractionation revealed that mitostatin is significantly enriched at inter-organelle microdomains referred to as mitochondrial-associated membranes (MAMs) [129]. MAMs are ultra-specialized synapses of endoplasmic reticulum (ER) with the outer mitochondrial membrane (OMM) that permits the bi-directional communication of ions and small chemical messengers that are critical for ER and mitochondrial homeostasis and overall cellular function [130]. Recent evidence has implicated MAM function as critical nodes necessary for mitophagic initiation by assembling signaling complexes, such as extracellular regulated kinase 2 (ERK2) [131] or PTEN-induced kinase 1 (PINK1) [132]. Among the many synaptic-like molecules found within MAMs, the primary component is the large, fusogenic GTPase known as mitofusin 2 [133], which is vital for maintaining mitochondrial function and morphology. Importantly, mitostatin physically interacts with the ectodomain of mitofusin 2 (MFN2) [129]. This interaction could modulate ER/mitochondrial tethering [134] in a mitostatin-dependent manner or could aid in recruiting pro-mitophagic components (such as the E3 ligase, Parkin [135]) to form a mitostatin/MFN2-positive signaling hub [136].

Concurrent with its effects on inhibiting migration, mitostatin over-expression severely disrupted the organization of the mitochondrial matrix resulting in disordered cristae architecture and triggered swelling, with affected mitochondria taking on a more stout and oblong morphology [127]. Mitostatin affects a molecular chaperone protein known as heat shock protein 27 (Hsp27), which has roles in modulating the mitochondrial-independent (extrinsic) apoptotic pathway [137] and actin re-organization [138,139]. Co-incident with these ultrastructural changes, mitostatin decreased Hsp27 phosphorylation at Ser82 (total Hsp27 levels remained unchanged) [127]. The biological role of decreased Hsp27 phosphorylation via mitostatin remains unknown; however, the mechanism behind this decrease and the functional connections it may have to modulating mitochondrial architecture following over-expression may be critical for its pro-mitophagic and anti-tumorigenic effects.

### 3.2. Mitostatin Is Necessary to Drive Decorin-Stimulated Breast Cancer Mitophagy

Proximal to decorin/MET binding is the first clear event during the initiation of the pro-mitophagic signaling cascade [25]. The master regulator of mitochondrial biogenesis and energy metabolism [140,141,142], peroxisome-proliferator activated receptor-α coactivator 1α (PGC-1α) is dynamically regulated in a spatiotemporal manner [76]. Strikingly, decorin triggers nuclear translocation of PGC-1α and directs it to directly bind *TCHP* mRNA via its C-terminal RNA recognition motif (RRM). This results in mitostatin protein to significantly accumulate [76]. Silencing PGC-1α or genetically deleting the RRM compromises *TCHP* mRNA stability and subsequently reduces the amount of cytosolic mitostatin.

Deciphering this cascade revealed an unlikely connection between mitostatin, a putative tumor suppressor gene, and PGC-1α, a known proto-oncogene that is necessary for mitochondrial biogenesis. Increased oxidative metabolism, via PGC-1α, MITF, and B-Raf [143] drives metastatic melanomas characterized by augmented mitochondrial respiratory capacity and oxidative stress resistance [144]. However, despite this oncogenic connection, this would not be the only instance of a cooperative loop to maintain proper mitochondrial homeostasis, which could be leveraged in breast cancer as a novel therapy. Further nuancing the intricate molecular complexity between decorin and PGC-1α is the role of AMPK in potentially transducing these signaling in TNBC. While it is known that decorin activates AMPK for autophagic induction in endothelial cells, it is unknown whether decorin stimulates AMPK in a similar manner. This would be intriguing, especially in light of how AMPK functions in TNBC via the activity of folliculin (FLCK) [145]. Folliculin has been characterized as a tumor-suppressor protein and forms a regulatory complex with AMPK [146]. The loss of FLCK results in the constitutive activation of AMPK, leading to enhanced engagement with PGC-1α, HIF-1α, and TFE3 to drive aggressive tumor formation and angiogenesis, particularly in TNBC [145]. Given this, decorin may finely regulate the interaction of FLCK with AMPK, and thus the output of AMPK signaling in TNBC to permit mitophagic activation and continued oncosuppression. This would be a key molecular interaction to investigate in endothelial cells where decorin does activate AMPK to drive the Peg3/TFEB axis for autophagic progression.

As discussed below, Parkin-mediated mitophagy is a major pathway to clear damaged and abnormal mitochondria. An elegant feedback loop centered around Parkin keeps the balance between mitophagy and mitochondrial biogenesis to ensure proper mitochondrial mass [147]. In this system, Parkin ubiquitinates components on the OMM for mitochondria destined for degradation via mitophagosomes while simultaneously targeting a transcription factor known as PARIS (ZNF746) for proteasomal degradation [148]. The loss of PARIS results in de-repressed PGC-1α (and its target nuclear respiratory factor 1, NRF1) to drive mitochondrial biogenesis to replace the mitochondria lost to mitophagy [147]. It is possible that mitostatin may be interfacing with PGC-1α in a similar manner to regulate the mitochondrial population. As a further layer of complexity underscoring this concept is that mitostatin binds Parkin following decorin stimulation (see below).

Mitostatin loss via RNAi abrogates basal and decorin-mediated mitophagy [149,150] including respiratory chain subunits, VDAC1, mitochondrial transcription factor A (TFAM), mtDNA, and mitochondrial network fragmentation [76] (Figure 2). Fragmentation of the mitochondrial network is a key step toward efficient mitophagy and is congruent with mitostatin over-expression [127]. Current studies are focusing on determining the role of decorin and mitostatin in driving mitophagic flux in an analogous mechanism for decorin and Peg3 to drive endothelial cell autophagic flux (see above).

**Figure 3:**
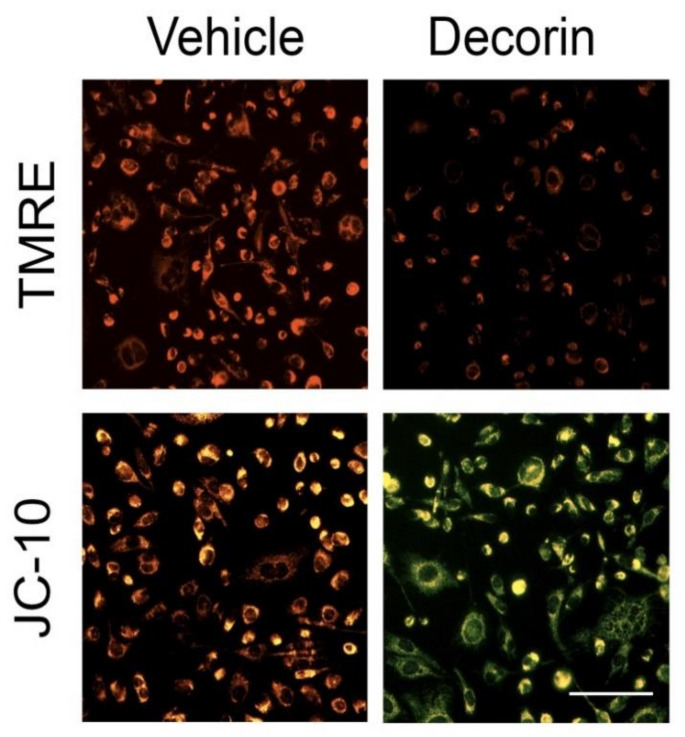
Representative live cell images depicting decorin-evoked mitochondrial depolarization in triple negative MDA-MB-231 cells using TMRE (top row) or JC-10 (bottom) relative to vehicle (PBS). The decorin protein core was used at 200 nM for 2 h. Upon depolarization, TMRE no longer accumulates within the mitochondrial matrix and the fluorescent signal fades; JC-10 no longer aggregates and thus undergoes a shift from red (JC-10 aggregates) to green (JC-10 monomers). Scale bar ~10 μm.

As an organellar harbinger of the mitophagy to come, decorin triggers rapid mitochondrial depolarization (∆Ψ_m_) (Figure 3) [76] as determined by staining with TMRE (Figure 3, top row) or JC-10 (Figure 3, bottom row). The magnitude of ∆Ψ_m_ is statistically comparable to (carbonyl cyanide-p-trifluoromethoxyphenylhydrazone) FCCP or carbonyl cyanide m-chlorophenylhydrazone (CCCP), which are established protonophores for the chemical uncoupling of the electron transport chain [151]. 

However, considering the vast differences in mechanisms between decorin (which is not cell permeable and therefore requires a membrane-bound RTK to signal) and FCCP (or CCCP) (membrane soluble), it stands to consider the downstream signaling complexes as active participants to transduce the ∆Ψ_m_ signal from the membrane to the mitochondrial machinery. Preliminary evidence, surprisingly, rules out the contribution from canonical kinases implicated in autophagic initiation, including Vps34 and AMPK (unpublished observations) in mediating ∆Ψ_m_. Importantly, this does not rule out AMPK at having vital roles in decorin-mediated mitophagy at later stages of the process [152]. Therefore, kinases, such as leucine rick repeat kinase 2 (LRRK2) [153], which localizes within MAMs [154] or mitochondrial localized ERK2 [131], are reasonable candidates to begin deciphering this important signaling mechanism.

As mitostatin localizes to the MAM and physically interacts with MFN2, it may permit a rapid efflux of ER Ca^2+^, perhaps via the large conductance inositol 1,4,50-triphosphate receptor (IP3R) activation, into the mitochondria to trigger mitophagy. Alternatively, decorin may have a role in reactive oxygen species (ROS) production, which is a potent activator of ∆Ψ_m_ [5,155]. Conceptually, this would place decorin as a ROS modulator, compounds already implicated as pro-mitophagic as therapy for breast cancer. The loss of ∆Ψ_m_ across the OMM is potent signal for PINK1/Parkin-mediated mitophagy [156,157,158,159]. Parkin is an RBR-domain containing E3-ubiquitin ligase commonly found within SCF-like ubiquitin ligase complexes [160] that is quickly recruited to the OMM following mitochondrial damage, such as loss of mitochondrial polarization. PINK1 is a mitochondrial-localized kinase that is protected from continued degradation [161] and thus accumulates upon the OMM [162] following ∆Ψ_m_ [132]. Recent evidence implicates an ECM connection where heparan sulfate structures can affect mitophagy in *D. melanogaster* Parkin models (see Section 4) [163].

Stabilized PINK1 phosphorylates multiple mitochondrial (VDAC1, translocase of the outer mitochondrial membrane, TOM) complexes [164] and cytosolic substrates, including ubiquitin (Ub) [165]. As it pertains to mitostatin biology, mitofusin 2 is a verified PINK1 substrate [166,167], whose phosphorylation is critical for culling damaged mitochondria via Parkin-mediated mitophagy [135]. It is unknown whether mitostatin contains a consensus PINK1 phosphorylation domain or if mitostatin, in response to decorin signaling, modulates PINK1 activity towards mitofusin 2. Phosphorylated Ub activates Parkin [168,169], which results in the generation of poly-Ub chains on key mitochondrial proteins [170]. Parkin then utilizes phospho-Ub to ubiquitinate several OMM components, including VDAC1 and p62 [171] following binding to dedicated Parkin receptors (Bnip3/Nix, FUNDC1, and NDP52 [132]). There is evidence that even the TOMM complex and/or VDAC1 [172], following PINK1-phoshorylation, serves as a Parkin receptor and subsequent signaling hub for Parkin-driven mitophagy [164] (Figure 2).

The recognition of the phospho-Ub substrates by various Ub-binding receptors (p62, optineurin, or NBR1) results in engulfment by LC3-positive autophagosomes and subsequent clearance [132]. Mechanistically, Parkin requires p62/VDAC1 binding for autophagosomal capture of selected mitochondria [171]. Parkin maintains mitochondrial homeostasis [160] and mitochondrial turnover in vivo [173], it is plausible that decorin directly recruits Parkin to the OMM following ∆Ψ_m_, in a mitostatin/mitofusin 2-dependent manner (Figure 2). It currently remains unknown if Peg3 is involved in this shuttling in a manner akin to VEGFA being shuttled into autophagosomes. Given the role of Peg3 as a tumor suppressor gene in breast cancer and its roles in autophagy, Peg3 may subsume an important regulatory function connecting mitostatin/Parkin to the mitophagy system.

This novel signaling pathway of decorin/MET/mitostatin/Parkin transduces signals from high affinity decorin/MET interactions via an unidentified kinase or similar effector, for sustained tumor cell mitophagy [174,175]. In line with our studies, it has been recently shown that mitostatin/Trichoplein binds pericentriolar material 1 protein (PCM1) and controls autophagy in endothelial cells [176]. Autophagy and mitophagy are emerging as the primary mechanisms of action that fully integrate and translate decorin/RTK antagonism across diverse tissues within the tumor into the established and classical anti-tumorigenic properties attributed to this proteoglycan.

## 4. A General Concept: Is Mitophagy Evoked by Other Secreted ECM Constituents?

We feel that the story with decorin and mitophagy may be the tip of the iceberg, that is, we predict that many more secreted ECM constituents would affect these intracellular catabolic pathways. For example, collagen VI is an abundant and ubiquitous ECM protein that is secreted by fibroblasts in all the major organs [177] and whose genetic defects are causatively linked to various mammalian congenital diseases [178]. Unexpectedly, *Col6a1*^−/−^ fibroblasts display abnormalities in the autophagy/lysosome machinery, impaired clearance of autophagosomes and failure of Parkin-dependent mitophagy [179,180]. Notably, adipocyte-derived collagen VI affects the early progression of mammary carcinomas in vivo, suggesting a critical role for this protein in the tumor microenvironment [181]. Another example of matrix-derived regulators of mitophagy is heparan sulfate, which appears to be a negative regulator of mitophagy. In *Drosophila Parkin* mutants, altering heparan sulfate biosynthesis suppresses mitochondrial dysmorphology indicating that the activation of mitophagy is potentiated in these mutants [182]. These findings suggest that a genetic background deficient in heparan sulfate, we do not know as of yet which heparan sulfate proteoglycan is involved in this process and attenuates the muscle phenotype in *Parkin* mutants, including restoration experiments [183].

There is also evidence that Irisin, a soluble peptide of 112 amino acids derived from the transmembrane protein called fibronectin type III domain containing protein 5 (FNDC5) [184], can positively affect mitophagy [185]. Indeed, Irisin mitigates oxidative stress and chondrocyte dysfunction through retaining mitochondrial biogenesis, dynamics, and autophagy [185] Moreover, Irisin is an exercise-induced myokine abundant in skeletal muscle and facilitates the positive impact of moderate exercise on tissue physiology and cognitive function [186,187]. Notably, PGC1-α activates FNDC5 to increase the secretion of Irisin [184] and, as mentioned above, decorin evokes mitophagy in breast carcinoma cells via PGC-1α and mitostatin [76]. Decorin has also been proposed to act as a myokine induced by exercise [188] and growth hormone [189]. Thus, it is possible that decorin and Irisin could be part of a network of secreted proteoglycans and proteins regulating mitophagy.

## 5. Conclusions: Challenges and Opportunities

The breakthrough discovery that selected proteoglycan family members are capable of potently and specifically regulating facets of intracellular catabolism, such as autophagy [73,105,190,191,192], represents a major conceptual and scientific advance for matrix biology. In particular, the soluble members of this cadre of proteins, represented first and foremost by decorin, are capable of evoking driving receptor tyrosine kinase-dependent autophagy by non-canonical means. Autophagic and mitophagic induction that is controlled by decorin requires a dedicated RTK (VEGFR2 or MET) and a dedicated tumor suppressor gene (Peg3 or mitostatin) to operate efficiently and optimally in different histological and morphological tissue compartments.

These decorin neofunctions [24], most of which are generalizable to the broader proteoglycan family, adds tremendous biological versatility and utility, while simultaneously expanding the known interactome [117] of these truly multifaceted proteins [28,73]. The kind of precise tissue specificity exhibited by decorin, conveyed by tissue specific RTK expression, could be leveraged therapeutically to target a particular pathway of interest. Indeed, delving deep into the mechanisms underlying how decorin regulates evolutionarily overserved processes make for attractive therapeutic targets [193,194].

Utilizing advanced, innovative, high-throughput, and high-resolution “-*omics*” approaches and emergent technologies, such as AI and machine-learning based systems, matrix biologists are currently expediting the full decoding the signaling pathways involved in such dynamic systems [195,196]. These approaches led to the identification of a master autophagic regulator, Peg3 [75] and the discovery of non-canonical, RTK-driven autophagy in normal endothelial cells, independent of prevailing nutrient conditions. This viewpoint was then extended and a corollary in breast cancer cells undergoing mitophagy was soon found, driven by an innate tumor suppressor gene, mitostatin. In both cases, decorin could tip the balance in favor of pro-autophagic/mitophagic signaling cascades, despite the layers of complex regulatory mechanisms and networks (mTOR vs. AMPK) governing cellular energy metabolism. Bypassing these systems permitted an excess level of autophagy or mitophagy to occurs, resulting in novel methods of angiogenic and tumorigenic inhibition.
